# Delta-9-Tetrahydrocannabinol (∆^9^-THC) Induce Neurogenesis and Improve Cognitive Performances of Male Sprague Dawley Rats

**DOI:** 10.1007/s12640-017-9806-x

**Published:** 2017-09-21

**Authors:** Noor Azuin Suliman, Che Norma Mat Taib, Mohamad Aris Mohd Moklas, Rusliza Basir

**Affiliations:** Department of Human Anatomy, Faculty of Medicine and Health Sciences, Universiti Putra Malaysia, 43400 Serdang, Selangor Malaysia

**Keywords:** Delta-9-tetrahydrocannabinol, Hippocampal neurogenesis, Cognitive function, Novel-object discrimination test

## Abstract

Neurogenesis is influenced by various external factors such as enriched environments. Some researchers had postulated that neurogenesis has contributed to the hippocampal learning and memory. This project was designed to observe the effect of Delta-9-tetrahydrocannabinol (∆^9^-THC) in cognitive performance that influenced by the neurogenesis. Different doses of ∆^9^-THC were used for observing the neurogenesis mechanism occurs in the hippocampus of rats. The brains were stained with antibodies, namely BrdU, glial fibrillary acidic protein (GFAP), nestin, doublecortin (DCX) and class III β-tubulin (TuJ-1). The cognitive test was used novel-object discrimination test (NOD) while the proteins involved, DCX and brain-derived neurotrophic factor (BDNF), were measured. Throughout this study, ∆^9^-THC enhanced the markers involved in all stages of neurogenesis mechanism. Simultaneously, the cognitive behaviour of rat also showed improvement in learning and memory functions observed in behavioural test and molecular perspective. Administration of ∆^9^-THC was observed to enhance the neurogenesis in the brain, especially in hippocampus thus improved the cognitive function of rats.

## Introduction

Delta-9-tetrahydrocannabinol (∆^9^-THC) is the main psychoactive substance that affects the mind or behaviour that mainly found in the *Cannabis sativa*, *C. indica* and *C. ruderalis*. ∆^9^-THC had been used for hemp, medical uses and as a recreational drug (Gaoni and Mechoulam 1964).

A number of studies had been conducted in order to observe the neurogenesis in hippocampus influenced by other substances (Eisch et al. 2000). Chronic treatment with a cannabinoid agonist such as Δ^9^-THC, altered the morphology of the hippocampus. The alterations included a decrease in the mean volume of neurons and also the number of synapses (Kim and Thayer 2001). Chronic treatment of Δ^9^-THC showed significant decreasing in the number of newly generated cells (Kochman et al. 2006).

Despite of improving the memory and learning performances of schizophrenia patient (Lieberman et al. 1987), there are lists of negative effects of ∆^9^-THC, such as euphoria, anxiety, and impairment of verbal working memory (D’Souza et al. 2004). Cognitive impairment was observed after heavy exposure (approximately 60 to 110 mg per day) and prolonged use of ∆^9^-THC presented as an unimpaired language function, memory, multimodal learning, and even intellectual functions (Schaeffer et al. 1981). Meanwhile, a study in 0.002 and 10 mg/kg of Δ^9^-THC showed impairment on the immediate learning and delay free remembering (D’Souza et al. 2005). In addition, the chronic users of cannabis were found to present non-acute effect of depletion of the capability to remember and learn new facts (Grant et al. 2003).

Through the reports, this study was done to observe the effect of Δ^9^-THC at low doses on hippocampal neurogenesis. Cognitive function was observed behavioural and molecularly.

## Materials and Methods

### Animals

Sixty-four male Spraque dawley rats weighted 200 to 300 g with averaged 4 weeks old were purchased from Faculty of Medicine and Health Sciences, Universiti Putra Malaysia (UPM). The study was reviewed by the Animal Care and Use Committee, Faculty of Medicine and Health Sciences, UPM. The approval number is UPM/FPSK/PADS/BR-UUH/00391.

### Treatment

Two batches of animals were used (*n* = 6), for Western blot and immunohistochemistry techniques. Three doses of ∆^9^-THC were selected (0.75, 1.5, and 3.0 mg/kg), together with negative control. The negative control was a vehicle used for ∆^9^-THC since 100% of ethanol was a stock solution of 100 mg/ml of the drug. The treatments were given intraperitoneally (i.p.) after 1 week of acclimatisation for 7 days (acute treatment) and 21 days (chronic treatment). On the last day of treatments, the rat was tested for cognitive performance using NOD test. The batch of animal for immunohistochemistry technique was sacrificed.

### Hippocampal Neurogenesis Study

#### BrdU Injection

The rats were injected with 50.0 mg/kg of BrdU (Roche, Germany), i.p., on the last day (Buga et al. 2012). The rats were decapitated a day after BrdU injection. The whole brain was removed and stored in 10% of formalin for 2 days. Then, the brain was processed and embedded in paraffin. The procedure of staining was run according to the manufacturer’s protocol.

#### Immunohistochemistry Technique

Paraffin-embedded sample was sectioned at a thickness of 10 μm on coated-slide (Poly-L-Lysine from Sigma, USA). The slide was dried at room temperature before the pretreatment procedure of deparafinization, rehydration and epitope retrieval in citrate buffer (pH 6.0) at 95 °C for 20–30 min. The slide was then left to cool down in citrate buffer (2.94 g of Tri-sodium dehydrate in 1 L of distilled water, pH 6.0, and 0.5 mL of Tween 20) at room temperature for 20 min, after that it was rinsed with the phosphate-buffered saline (PBS) Tween 20 (3 min, 2×). Then, the slide was incubated in 5% BSA in PBS at room temperature for 30 min, and again incubated with primary antibody (4 °C for overnight) for DCX, TuJ-1, GFAP and Nestin (Abcam, USA).

The slide was rinsed in PBS Tween 20 followed by incubation with 3% H_2_O_2_ in water at room temperature for 30 min, rinsed in PBS Tween 20 and followed by incubation with secondary antibody at room temperature for 30 min (Abcam, USA). The slide was then rinsed with PBS Tween 20, and again incubated with 0.05% DAB in Tris-buffered saline (TBS) for 15 min and rinsed in PBS Tween 20. The counterstain was done using H&E staining.

#### Analysis

The slide was viewed using an Olympus BX51 and the images of the hippocampus were captured and saved using Olympus Cell^F imaging software. The number of positive-stained cells of each ×400 magnification hippocampus area was counted manually (Armstrong et al. 2004). Data was analysed using SPSS 16.0 and Kruskall-Wallis test was used. Significant differences among the treatments and dosages was determined by the *p* value where *p* < 0.05 (*). Data presented as mean ± standard error of mean (± S.E.M).

### Cognitive Perspective

#### Behavioural Test

**Fig. 1:**
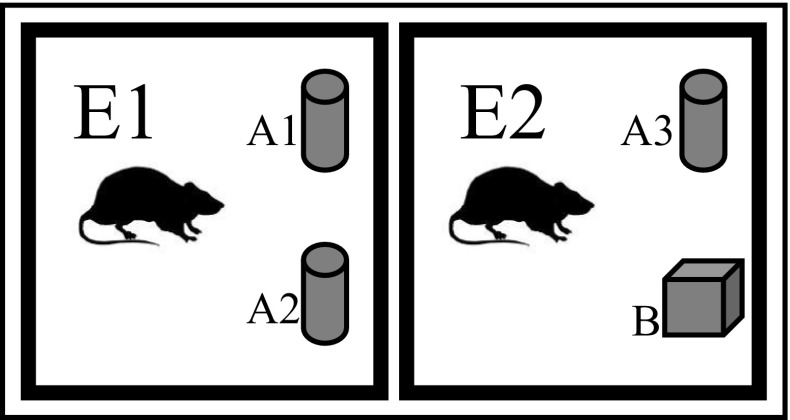
Novel-object discrimination test (NOD) layout on the Perspex box. The diagram shows the arrangement of the rat, familiar objects (A1, A2, A3), and novel object (B) in the perspex box during first exposure (E1) and second exposure (E2)

The rat was acclimatised in the behaviour room for 1 h prior to test (Bevins and Besheer 2006). The rat was placed in the centre of a perspex box (Fig. 1) for 5 min. The interval time between first (E1) and second (E2) exposures for short- and long-term memories working were 1 min and 1 day, respectively. The rat was placed in the perspex box which contains familiar and novel objects for 5 min. Time spent on each object (A3 and B) was recorded. The behaviour of rat was monitored from other room that equipped with camera and closed-circuit television (CCTV). The exploration of the rat was presented by distance of the rat’s nose was less than 2 cm to the object and/or touching it. The discrimination ratio (D1) was a difference between the B and A3 while the discrimination index (D2) was calculated by dividing the D1 to the total of B and A3 (Bevins and Besheer 2006).

#### Protein Determination

##### Western Blot

A day after behavioural test, the rat was sacrificed through decapitation and hippocampus was quickly collected and stored at − 70 °C prior to use. The hippocampus was homogenised in sucrose lysis (pH 7.8), protease inhibitor (Roche, Germany) and phosphatase inhibitor (Sigma, USA). The samples were centrifuged at 13000 rpm for 10 min at 4 °C. Cytosolic fraction was aspirated for total protein determination using Bradford reagent and further being aliquot at 1 mg/mL using sucrose lysis. A 1 mg/ml sample were run for SDS-PAGE electrophoresis and transferred onto PVDF membrane. The blot was probed with the primary antibody, DCX (Cell Signalling Technology, USA) and β-actin (Abcam, USA), overnight at 4 °C and subsequently incubated with horseradish peroxidise-conjugated secondary antibody (Dako, USA) for 1 h. Immunoreactive protein was detected by a chemiluminescent method (Thermo Scientific, USA) according to the manufacturer’s protocol and viewed using gel documentation (INFINITY system, Vilber Lourmat, Germany). The image obtained was measured using ImageJ software. The data were calculated following Long’s report (2010).

##### ELISA Technique

The rat was dissected through decapitation whereby the hippocampus was separated and stored in − 80 °C prior to use. The brain was homogenised using iced-cold phosphate buffer saline at pH 7.0 at ratio 1:100. The sample was centrifuged at 3000×*g* for 10 min. The supernatant was removed and aliquoted for further used. The sample was run for detecting BDNF using an ELISA kit (Product no. SEA011Rb, USCNK) according to the manufacturer’s protocol.

#### Analysis

Discrimination index (D2) and protein expression were analysed using SPSS 21.0 for one-way analysis of variance (ANOVA) analysis and followed by Tukey’s multiple comparison test. Significant differences among the treatments and dosages were determined by the *p* < 0.05 (*). Data was presented as mean ± standard error of mean (S.E.M.).

## Results

### Hippocampal Neurogenesis Study

**Fig. 2:**
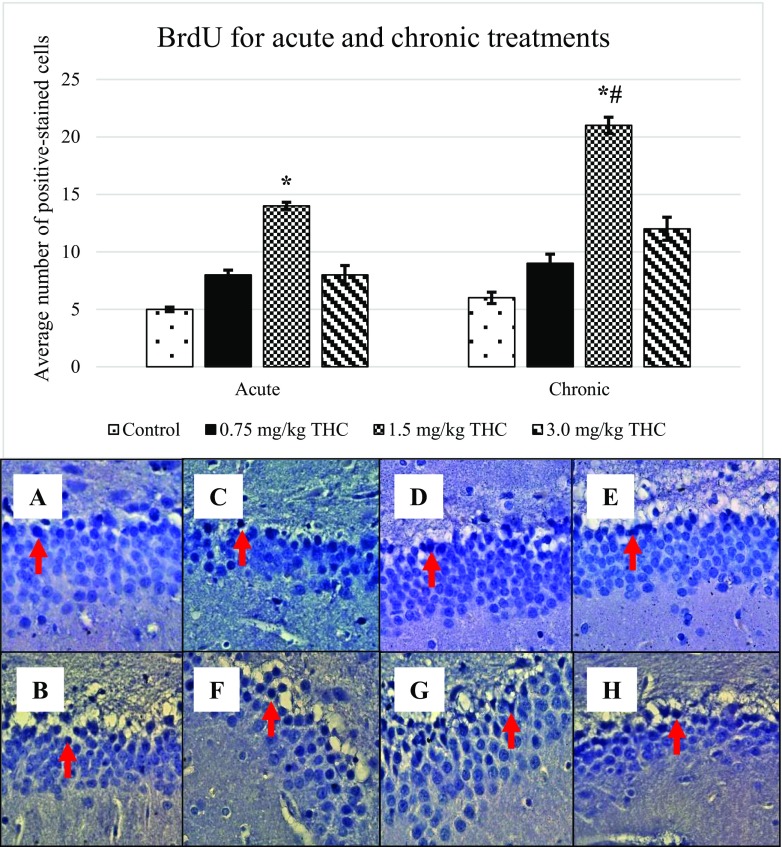
Expression of bromodeoxyuridine (BrdU). BrdU-positive cell appears blackish in colour, as presented in the arrow above. BrdU-positive cell appears in the GCL in the DG. Pictures of **a** and **b** show the positive cell of control, **c**–**e** are for acute treatment, while **f**–**h** are for chronic treatment

Injection of BrdU was performed to observe the presence of newborn cells including neuron cells. Figure 2 showed the average number of BrdU-positive cell at magnification of ×400 (± S.E.M) for both acute and chronic treatments. Significant differences were observed at both treatments against control and dosages of ∆^9^-THC at *p* < 0.05. Control, 0.75 and 3.0 mg/kg of ∆^9^-THC were noticeably different compared to 1.5 mg/kg of ∆^9^-THC at *p* < 0.05 (*). Chronic treatment of 0.75, 1.5 and 3.0 mg/kg of ∆^9^-THC showed significant differences as compared to acute treatment of the same doses (^#^ *p* < 0.05) while the control of chronic treatment was comparable to acute.

**Fig. 3:**
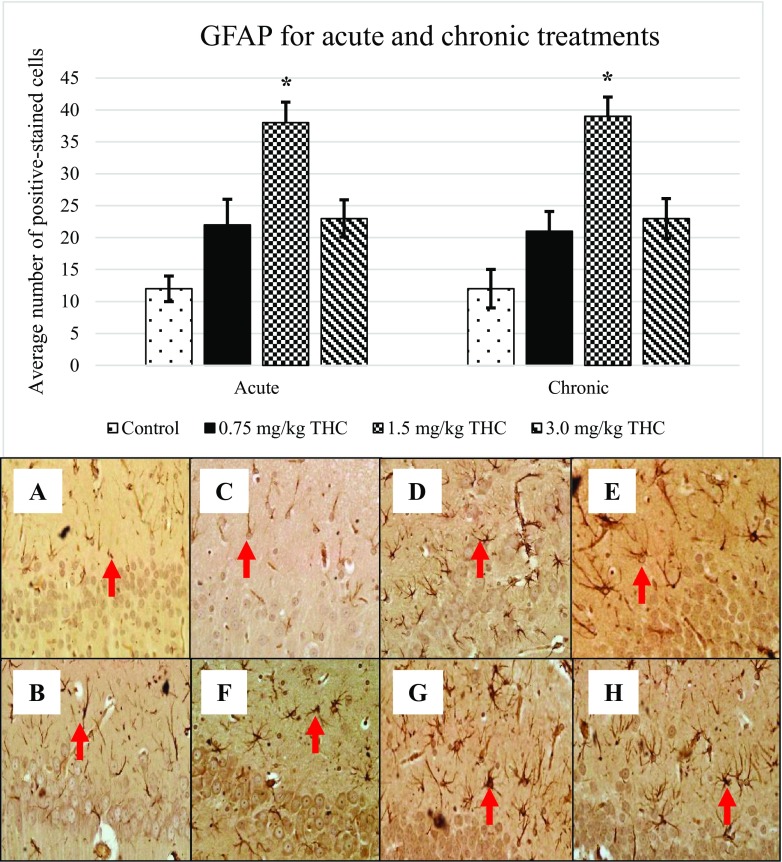
Upregulation of glial fibrillary acidic protein (GFAP). Arrows above show the GFAP-positive cell that appeared brownish in colour. GFAP-positive cell presents in the GCL in the DG. Pictures of **a** and **b** show the positive cell of control, **c**–**e** are for acute treatment, while **f**–**h** are for chronic treatment

**Fig. 4:**
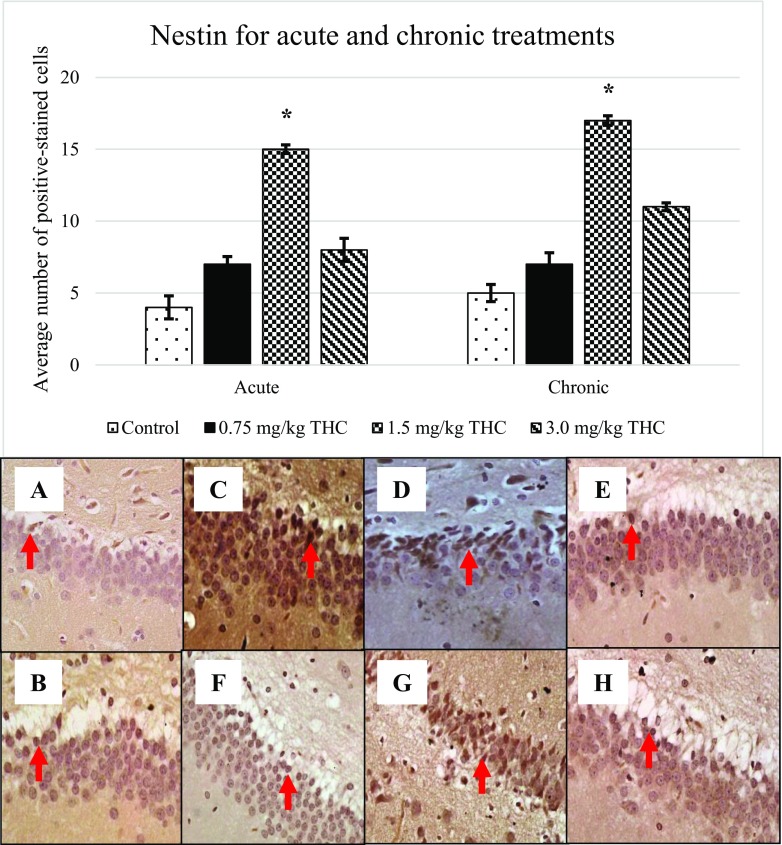
Appearance of nestin. Arrows above show the nestin-positive cell that appeared brownish in colour. Pictures of **a** and **b** show the positive cell of control, **c**–**e** are for acute treatment, while **f**–**h** are for chronic treatment

**Fig. 5:**
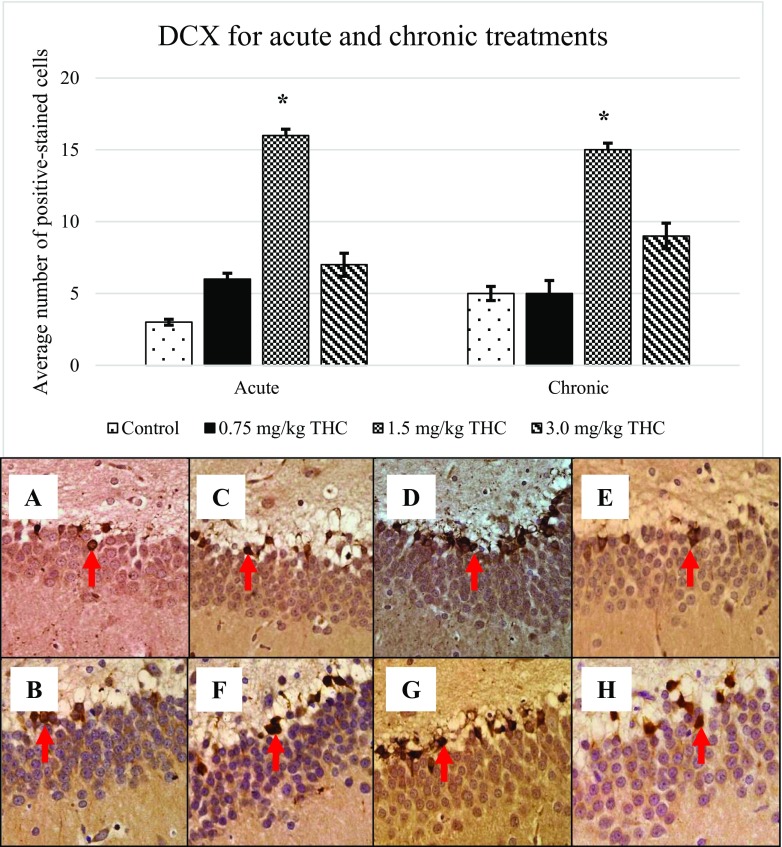
Upregulation of doublecortin (DCX). Arrows above show the DCX-positive cell that appeared brownish in colour. Pictures of **a** and **b** show the positive cell of control, **c**–**e** are for acute treatment, while **f**–**h** are for chronic treatment

**Fig. 6:**
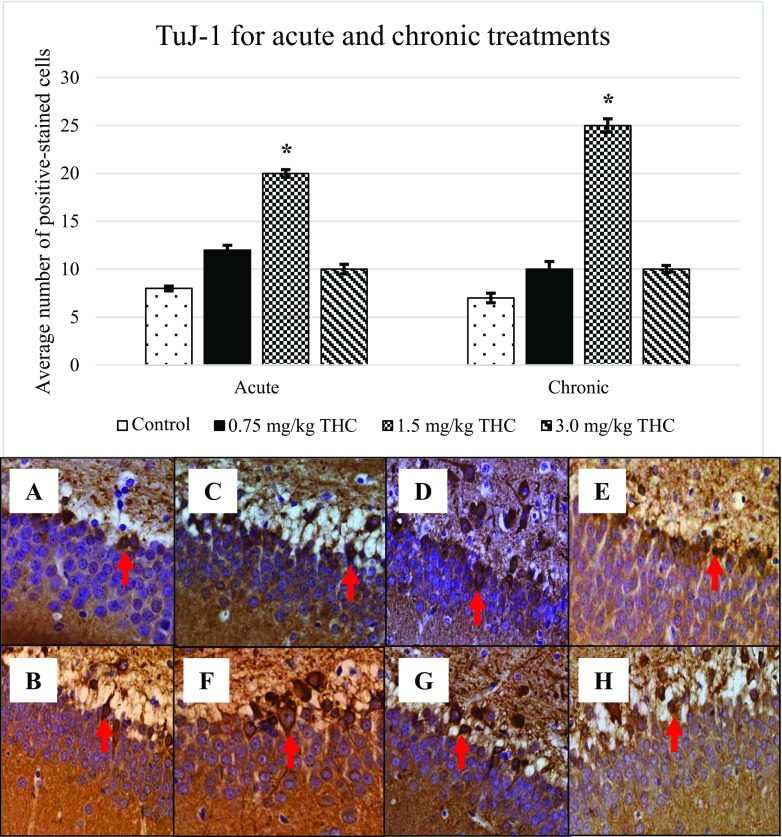
Increment of class III β-tubulin (TuJ-1). Arrows above show the TuJ-1-positive cell that appeared brownish in colour. Pictures of **a** and **b** show the positive cell of control, **c**–**e** are for acute treatment, while **f**–**h** are for chronic treatment

Following BrdU expression, other markers; GFAP (Fig. 3), nestin (Fig. 4), DCX (Fig. 5) and TuJ-1 (Fig. 6) that were involved in neurogenesis mechanism were performed. Expression of nestin, DCX and TuJ-1 were observed to express significant difference by comparing the control, 0.75, and 3.0 mg/kg of ∆^9^-THC to 1.5 mg/kg of ∆^9^-THC (*p < 0.05) (Figs. 3, 4, 5 and 6). Expression of the markers showed a similar pattern of induction whereby the highest number of positive-stained cell expressed in the rat treated with 1.5 mg/kg of ∆^9^-THC (*p* < 0.05). The overall induction of markers was simplified in Table 1.

**Table 1:** Simplified data for hippocampal neurogenesis study and cognitive perspective

Hippocampal neurogenesis study					
Marker	Treatment	Control	0.75 mg/kg THC	1.5 mg/kg THC	3.0 mg/kg THC
BrdU	Acute	N	N	↑↑	N
	Chronic	N	N	↑↑↑	↑
GFAP	Acute	N	↑	↑↑	↑
	Chronic	N	↑	↑↑	↑
Nestin	Acute	N	N	↑↑	↑
	Chronic	N	N	↑↑	↑
DCX	Acute	N	N	↑↑	N
	Chronic	N	N	↑↑	↑
TuJ-1	Acute	N	N	↑↑	N
	Chronic	N	N	↑↑	N
Cognitive perspective					
Behavioural test	Short-term				
	Acute	N	N	↑↑	↑
	Chronic	N	N	↑↑	↑
	Long-term				
	Acute	N	↑	↑↑	↑
	Chronic	N	↑	↑↑	↑↑
DCX	Acute	N	N	↑↑	N
	Chronic	N	N	↑↑↑	N
BDNF	Acute	N	N	↑↑	N
	Chronic	N	N	↑↑	N

Abbreviations: *BrdU*, bromodeoxyuridine; *DCX*, doublecortin; *TuJ-1*, class III β-tubulin; *GFAP*, glial fibrillary acidic protein; *BDNF*, brain-derived neurotropic factor; *N*, normal observation; *Upward arrow*, increase of expression

*p < 0.05 vs control, 0.75, and 3.0 mg/kg of THC; ^#^ *p* < 0.05 vs acute treatment

### Cognitive Perspective

#### Behavioural Test

**Fig. 7:**
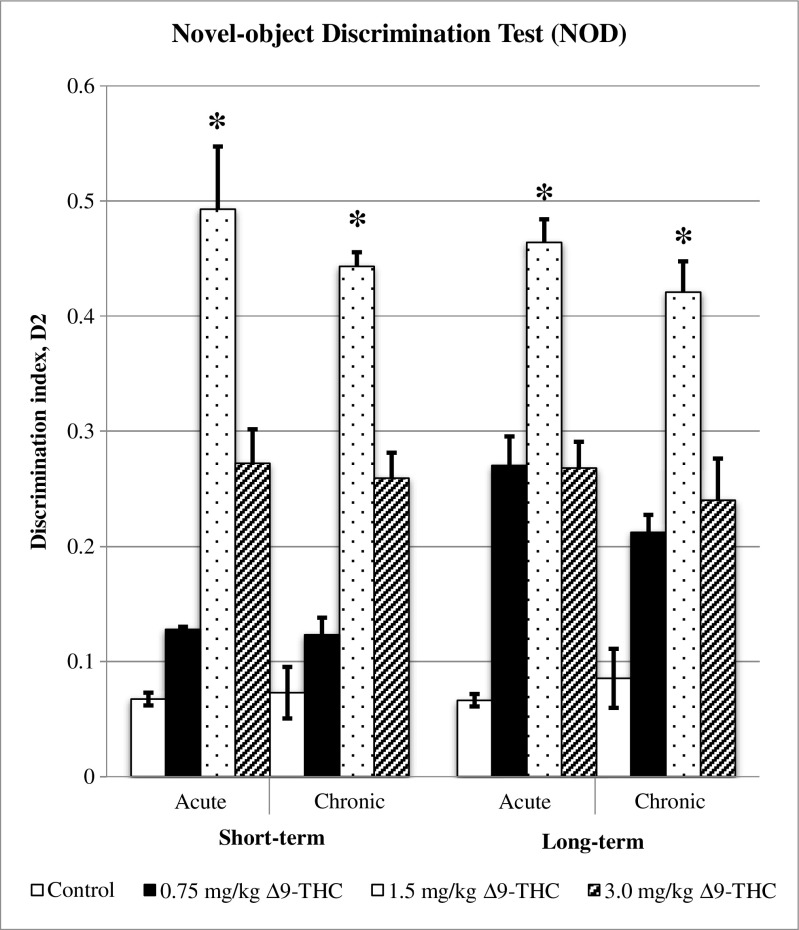
Discrimination index (D2). Graph shows the discrimination index (± S.E.M.) for acute and chronic treatments, both for short- and long-term memories. Increasing D2 indicates the increasing in time spent on novel object (B), suggesting the improvement of cognitive performance. There are significant differences between the control, 0.5, and 3.0 mg/kg of Δ^9^-THC as compared to 1.5 mg/kg of Δ^9^-THC (*p < 0.05). The D2 for acute treatment is comparable to chronic, applied for short- and long-term memories. *p < 0.05 vs 1.5 mg/kg of Δ^9^-THC

Discrimination index (D2) was calculated from D1 data over total time spent on both A3 and B objects. Figure 7 illustrates the data of D2 (± S.E.M) for short- and long-term memories, acute and chronic treatments. In short-term memory, acute and chronic treatments of 1.5 mg/kg of ∆^9^-THC showed significant differences (*p < 0.05) in recognising the novel object from familiar one as compared to other doses of ∆^9^-THC and control groups. Meanwhile, 3.0 mg/kg of ∆^9^-THC increased the D2, significantly difference as compared to 0.75 and 3.0 mg/kg of ∆^9^-THC (*p* < 0.05), applied for both acute and chronic treatments. Acute treatment was observed to be comparable to chronic treatment, comparing the respective doses.

As for long-term memory, 1.5 mg/kg of ∆^9^-THC showed significant difference as compared to control, 0.75, and 3.0 mg/kg of ∆^9^-THC (*p < 0.05). The treatments of 0.75 and 3.0 mg/kg of ∆^9^-THC showed significant differences compared to control (*p* < 0.05), applied for acute and chronic treatments.

#### Protein Determination

**Fig. 8:**
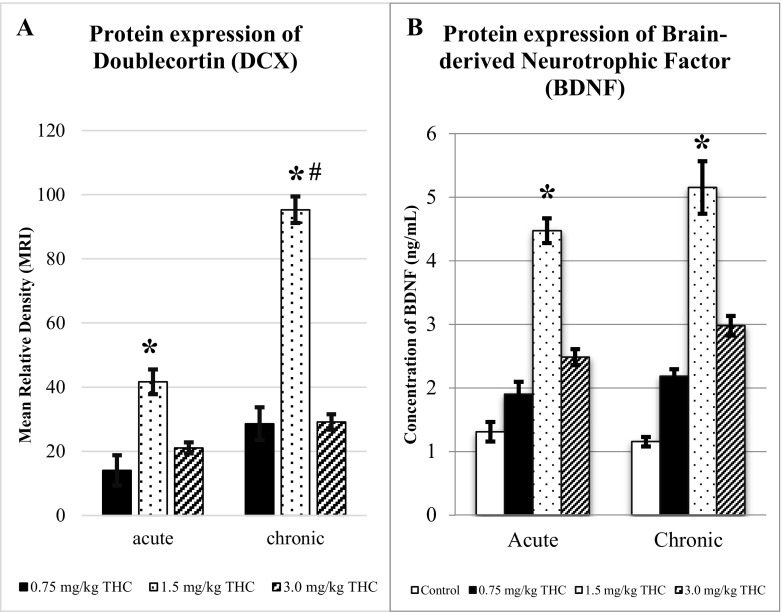
Doublecortin (DCX) and brain-derived neurotrophic factor (BDNF). Graphs shows the expression of doublecortin (DCX) (**a**) and brain-derived neurotrophic factor (BDNF) (**b**) for acute and chronic treatments (± S.E.M.). Increment expression of both marker suggesting neurogenesis and cognitive enhancement affected by ∆^9^-THC. The expression of DCX and BDNF are increased as compared to control. There are significant differences between the control, 0.5, and 3.0 mg/kg of Δ^9^-THC as compared to 1.5 mg/kg of Δ^9^-THC (*p < 0.05). Chronic treatment of 1.5 mg/kg of Δ^9^-THC is significantly difference as compared to acute treatment of the same dose (^#^ *p* < 0.05). The expression of BDNF for acute treatment is comparable to chronic. *p < 0.05 vs 1.5 mg/kg of Δ^9^-THC

Figure 8 showed the data for DCX (graph A) and BDNF (graph B) for both acute and chronic treatments. Data for DCX was represented by MRI (± S.E.M) while BDNF in unit of nanograms per millilitre. There were significant differences between 0.75 and 3.0 mg/kg of ∆^9^-THC to 1.5 mg/kg of ∆^9^-THC (*p < 0.05) for both acute and chronic treatments. The chronic treatment of 1.5 mg/kg of ∆^9^-THC was significantly difference as compared to acute treatment (^#^ *p* < 0.05). There was no significant difference between 0.75 and 3.0 mg/kg of ∆^9^-THC, applied for both acute and chronic treatments. Increasing calculated MRI of ∆^9^-THC-treated rat showed the upregulation of DCX expression on the hippocampus as compared to that of control-treated rat.

As for BDNF, similar patterns of observations to DCX were observed. The treatment of 1.5 mg/kg of ∆^9^-THC upregulated the expression of protein as compared to control, 0.75 and 3.0 mg/kg of ∆^9^-THC (*p* < 0.05), both in acute and chronic treatments. The treatment of control, 0.75, and 3.0 mg/kg of ∆^9^-THC showed no significant differences, applied for both acute and chronic treatments. The chronic treatment of all doses was comparable to acute treatment by comparing respective doses.

By comparing the behavioural test and proteins expression, there was similar increment by the 1.5 mg/kg of ∆^9^-THC. The rat treated with 1.5 mg/kg of ∆^9^-THC showed an increased D2, consistent with highest expression of DCX and BDNF, as compared to control, 0.5, and 3.0 mg/kg of ∆^9^-THC. Table 1 showed a simplified the data of the cognitive perspective.

## Discussion

Normal adult neurogenesis is known to occur in the brain, specifically in the hippocampus presented by the proliferation of cells. Production of new neurons in the dentate gyrus (DG) of hippocampus is related to the learning and memory functions (Shors et al. 2002). Introduction of ∆^9^-THC or other cannabinoid drugs affect the adult neurogenesis and later influence the cognitive function (Jiang et al. 2005).

Neurogenesis mechanism had been illustrated in six developmental milestone, as described by Kempermann et al. (2004). Meanwhile, Von Bohlen und Halbach (2007) had simplified the mechanism into five stages, namely proliferation (nestin), differentiation (nestin, Pax6), migration (NeuroD, DCX, PSA-NCAM), axonal and dendritic targeting (PSA-NCAM, DCX, TUC-4, Calretinin), and synaptic integration (NeuN, TuJ-1, Calbindin). As proposed by von Bohlen und Halbach (2007), the expression of markers is observed in different time point to describe the different stages of neurogenesis. However, due to limited information of altered cognitive function by ∆^9^-THC-induced neurogenesis, the expression of markers was studied in one time point.

Throughout the study, expression of BrdU, GFAP, nestin, DCX and TuJ-1 in ∆^9^-THC-treated rats were increased as compared to that in control, supported by Kaplan and Bell (1984). Kaplan and Bell (1984) had demonstrating an adult neurogenesis in the hippocampus. Reflecting the review by von Bohlen und Halbach (2007), studied markers are responsible for neurogenesis.

BrdU is a thymidine analogue that was suggesting to incorporate with the deoxyribonucleic acid (DNA) of dividing cells during the S-phase of the cell cycle as it is used in monitoring the cell proliferation occurs in the tissues (Philippe 2006). BrdU is used to confirm the division of neuronal progenitor cell. The BrdU-positive cell can be observed at the border between hilus and the granule cell layer (GCL) of DG (Scott et al. 1998). As BrdU labelled all S-phase cells, the labelled cells were indifferent between newly formed glia cells and neurons. Without the use of other neurogenesis markers, BrdU labelling demonstrating the general cell genesis occurs in the brain (von Bohlen und Halbach 2007).

Present data showed the upregulation of nestin at all doses of ∆^9^-THC. It was suggested these markers expressed on the proliferative neuron stage (von Bohlen und Halbach 2007). In differentiation phase, double-stained cell was expressed with nestin-positive and GFAP-negative. Later, the cell will stop expressing nestin while start to express doublecortin (DCX) and polysialylated embryonic form of the neural cell adhesion molecule (PSA-NCAM) (Fukuda et al. 2003). Increased DCX after the acute and chronic treatments of ∆^9^-THC in the study was corresponding to differentiation and migration stages of neuron. Meanwhile, elevated expression of TuJ-1 upon the treatment of ∆^9^-THC was reflecting the survival or differentiation of neuron (von Bohlen und Halbach 2007).

**Fig. 9:**
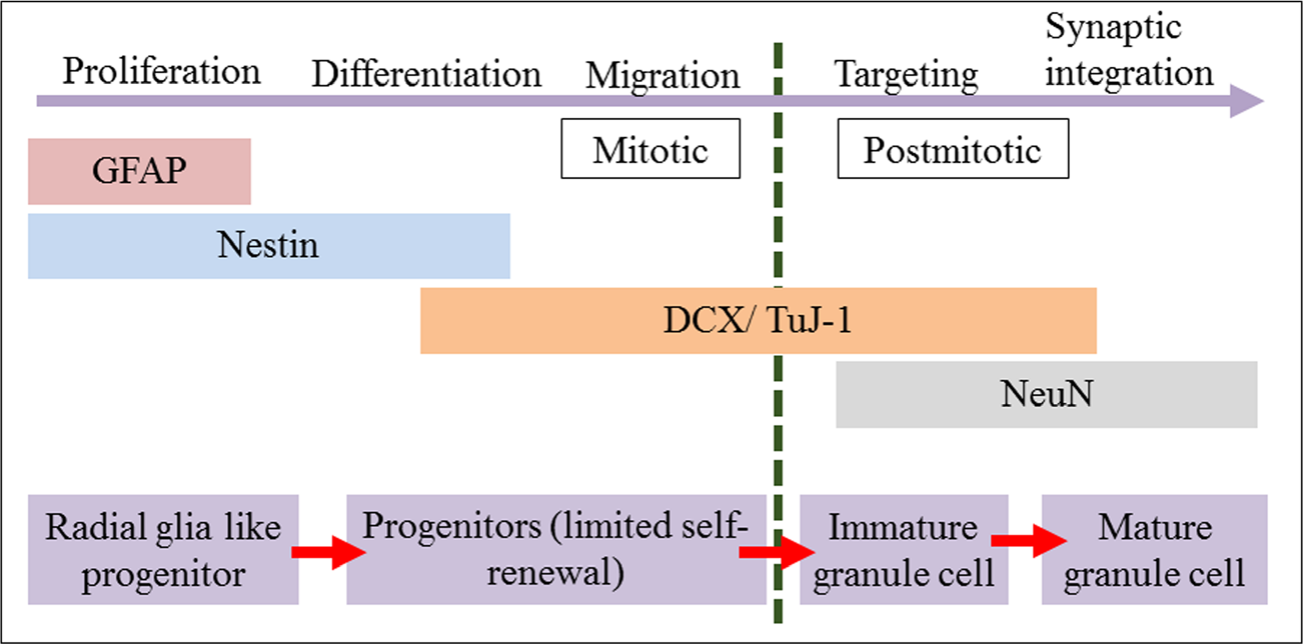
Illustrated mechanism of neurogenesis (DCX, doublecortin; TuJ-1, class III β-tubulin; GFAP, glial fibrillary acidic protein). The figure showed the stages of adult neurogenesis in the dentate gyrus and the expression pattern of the specific markers. (Source: von Bohlen und Halbach 2007).

Throughout the study, there are consistent findings for all the markers involved in the neurogenesis process in the rat treated with ∆^9^-THC. Although the present research was only used one time point, the mechanism of neurogenesis was unclear, there is illustrated process extracted from this study. The consistent expression of all markers as simplified in Table 1 has exemplified the effect of ∆^9^-THC on the respected markers as illustrated in Fig. 9.

As reported by Nyffeler et al. (2010), the activity of neurogenesis has improved the cognitive performance. To observe the interaction of interaction of curiosity and memory, NOD test was used to represent the declarative memory as it gave a few exposures to the animals as cues to learn. In natural exploratory behaviour, normal animals tend to increase the exploratory behaviour to novel stimuli and decrease the exploratory behaviour on familiar stimuli (Shors et al. 2002). The ∆^9^-THC-treated rat noticeably distinguished the familiar and novel object by spending lesser time on the familiar object compared to control-treated rat. The acute and chronic treatments of 1.5 mg/kg of ∆^9^-THC showed significant improvement studied by the discrimination index, D2. The rat treated with ∆^9^-THC spent more time on the novel object, postulating the rat was able to recognise the familiar object introduced before. The ability of rat to differentiate the objects reflecting the ability to learn and memorise the previous experiences (Bevins and Besheer 2006) and the similar pattern of data were observed in short- and long-term memory tests for acute and chronic treatments.

Involvement of DCX in the cognitive perspective was related to the neurogenesis (Nyffeler et al. 2010). Neurogenesis activity was reported to increase after the cognitive task demonstrated by the up-regulation of DCX expression. Beside cognitive tasks, enrichment of the environment and exercise were suggested to cause increment in the expression of DCX that was explaining the neurogenesis activity. Expressions of DCX at DG strengthen the postulation of learning and memory function (Barnea and Nottebohm 1994). Present data showed the increased expression of DCX in the ∆^9^-THC groups. The positive value of mean relative density was reflecting the increased expression of the protein as compared to that in control (Long et al. 2010). In response to cognitive test done before, the learning and memory activities had increase neuron plasticity expressed by the expression of DCX (Nyffeler et al. 2010). Although cognitive test did not outline the significant different between the acute and chronic treatments, expression of DCX showed significant increased level of DCX in chronic treatment as compared to that in acute. The chronic treatment of 1.5 mg/kg of ∆^9^-THC was elevating the level of DCX significantly as compared to acute treatment.

Expression of BDNF in this study proposed its roles in memory and learning leading to the process of hippocampal long-term potential (LTP) (Tyler 2002). BDNF is a member of the neurotrophin family that plays a role in neuronal survival, differentiation and synaptic plasticity (Lu et al. 2008). Previous experience on the learning and memory had increased the expression of BDNF. Present data showed the acute and chronic treatments of ∆^9^-THC increased the level of BDNF as compared to control. Increased plasticity activity through behavioural experience had increased the plasticity markers, BDNF and DCX supported by the Gooney’s report (Gooney et al. 2002).

The presented data were contrast to the report by Kim and Thayer (2001). Instead of using animal, Kim and Thayer (2001) used cultured hippocampus derived from rat. They demonstrated the inhibition of synapses and newly generated cells by exposing the forskolin-induced cell with ∆^9^-THC. The present study observed the increased neurogenesis by inducing the plasticity activity of the neuron. The flaw of this study was lacking study on functional synaptic boutons. As reported by Gooney et al. (2002) and Lu et al. (2008), learning and memory activities leading to plasticity of the neuron, thus leading to adult neurogenesis to take place.

Brain plasticity is modulated by the endocannabinoid system, involving CB1 and CB2 receptor. The consumption of ∆^9^-THC as synthetic cannabinoid has described on the neuronal progenitor cells. Wolf et al. (2010) has elucidated the involvement of CB1 in the adult neurogenesis. However, the study has reported the cognitive impairment accompanied with the treatment of ∆^9^-THC, which was contrast with present study. Taken together by other reports, ∆^9^-THC cannot be plainly categorised into impairing or enhancing (Abush and Akirav 2010), indeed it requires more research considering the route of administration, region of brain affected (Lorivel and Hilber 2007), and behavioural test used (Wolf et al. 2010). Although the present study did not elaborate on the receptor involved, many reports have elucidate the involvement of CB1 in the neurogenesis and cognition functions, taking into account the abundant volume of CB1 in the brain as compared to CB2 (Jin et al. 2004). The inhibition of CB1 had been reported to decrease the rate of neurogenesis process (Jin et al. 2004; Kim et al. 2006).

The study had demonstrated the influenced of ∆^9^-THC on the cognitive performances of the rat by increasing the learning and memory functions accompanied by high expression of plasticity markers, DCX and BDNF. The adult neurogenesis was demonstrated by the upregulation of BrdU, nestin, TuJ-1 and DCX. The acute and chronic treatments of ∆^9^-THC had increased the cognitive function of Sprague Dawley, demonstrated by behavioural and molecular perspectives. Table 1 has simplified the data for neurogenesis and cognitive perspectives. The treatment of 1.5 mg/kg of ∆^9^-THC has increase all the markers for neurogenesis and cognition function while improve the cognitive performance.

Neuron plasticity by the learning and memory activities had stimulate the adult neurogenesis to take place in the brain of the rat. Administration of ∆^9^-THC as synthetic cannabinoid was observed to enrich the neurogenesis process while influence the cognitive performance of rat dose-dependently. It was consistent by Jiang et al. (2005), reported the role of cannabis in cognitive functions.
